# Neuroligin 2 R215H Mutant Mice Manifest Anxiety, Increased Prepulse Inhibition, and Impaired Spatial Learning and Memory

**DOI:** 10.3389/fpsyt.2017.00257

**Published:** 2017-11-27

**Authors:** Chia-Hsiang Chen, Pin-Wei Lee, Hsiao-Mei Liao, Pi-Kai Chang

**Affiliations:** ^1^Department of Psychiatry, Chang Gung Memorial Hospital-Linkou, Taoyuan, Taiwan; ^2^Department and Graduate Institute of Biomedical Sciences, Chang Gung University, Taoyuan, Taiwan; ^3^Center for Biologics Evaluation and Research, Food and Drug Administration, Silver Spring, MD, United States; ^4^Graduate Institute of Biomedical Sciences, Department of Physiology and Pharmacology, Chang Gung University, Taoyuan, Taiwan

**Keywords:** neuroligin 2, schizophrenia, mutation, transgenic mice, anxiety, memory

## Abstract

Neuroligin 2 (*NLGN2*) is a postsynaptic adhesion protein that plays an essential role in synaptogenesis and function of inhibitory neuron. We previously identified a missense mutation R215H of the *NLGN2* in a patient with schizophrenia. This missense mutation was shown to be pathogenic in several cell-based assays. The objective of this study was to better understand the behavioral consequences of this mutation *in vivo*. We generated a line of transgenic mice carrying this mutation using a recombinant-based method. The mice were subjected to a battery of behavioral tests including open field locomotor activity assay, prepulse inhibition (PPI) assay, accelerated rotarod test, novel location and novel recognition tests, elevated plus-maze (EPM) test, and Morris water maze test. The transgenic animals were viable and fertile, but the *Nlgn2* R215H knock-in (KI) homozygous mice showed growth retardation, anxiety-like behavior, increased PPI, and impaired spatial learning and memory. There was no significant interaction between sex and genotype in most behavioral tests; however, we observed a significant interaction between sex and genotype in EPM test in this study. Also, we found that the *Nlgn2* R215H homozygous KI mice did not express the *NLGN2* protein, resembling *Nlgn2* knockout mice. Our results demonstrate that *Nlgn2* R215H KI homozygous mice manifest several behavioral abnormalities similar to those found in psychiatric patients carrying *NLGN2* mutations, indicating that dysfunction of *NLGN2* contributes to the pathogenesis of certain psychiatric symptoms commonly present in various mental disorders, not limited to schizophrenia.

## Introduction

Neuroligins are a family of postsynaptic adhesion proteins that interact with presynaptic neurexins to bridge the synaptic cleft (1). Neuroligins play an important role in the synaptic function and plasticity in the brain. Their proper function contributes to the formation of excitatory and inhibitory neurons and facilitates synaptic signal transmission (2–4). There are five genes encoding the neuroligin protein family in human, including neuroligin 1 (*NLGN1*), neuroligin 2 (*NLGN2*), neuroligin 3 (*NLGN3*), neuroligin 4, X-linked (*NLGN4X*), and neuroligin 4, Y-linked (*NLGN4Y*). *NLGN1* protein is located at excitatory postsynaptic membranes (5), *NLGN2* protein is selectively found at inhibitory postsynaptic membranes (6, 7), and nlgn3 protein is present at both excitatory and inhibitory postsynaptic membranes (8). Neuroligin 4 is present in glycinergic postsynaptic membranes (9).

Mutations in the neuroligin gene family have been detected in patients with various neuropsychiatric disorders, including autism, mental retardation, schizophrenia, and other cognitive disorders. For example, mutations in the *NLGN3* and *NLGN4X* genes were detected in patients with autism spectrum disorders and mental retardation (2, 10–13). A chromosomal loss of 3q26.3–3q26.32 that involves a partial *NLGN1* deletion was identified in a child with microcephaly, seizure disorder, and severe intellectual disability (14). Recently, a nonsense mutation of *NLGN1* that leads to truncated protein was detected in a patient with Alzheimer’s disease (15). Genetic polymorphisms of *NLGN1* were reported to be associated with schizophrenia (16) and posttraumatic stress disorder (17). In addition, a nonsense mutation of *NLGN2* was detected recently in a young male patient manifesting severe anxiety, obsessive-compulsive behaviors, autism, intellectual disability, and other aberrant developmental features (18). Together, these findings suggest that mutations of the neuroligin gene family are part of the genetic underpinnings of neurodevelopmental disorders, and provide evidence to support that synaptic dysfunction contributes to the pathogenesis of certain neuropsychiatric disorders (19, 20).

In our previous study, we systematically screened for mutations in exon and promoter regions of *NLGN2* in a sample of patients with schizophrenia. We identified six rare missense point mutations in this sample, including R215H, V510M, R621H, A637T, P800L, and A819S. Among these missense mutations, we found that R215H mutation was a loss-of-function mutation. It was shown to cause impairment in heterophilic cell adhesion, a deficit in presynaptic differentiation, and defective synaptogenic function in various cell-based assays (21), suggesting that the R215H is a pathogenic mutation of schizophrenia.

Prompted by these findings from *in vitro* studies of R215H, we were interested to understand the behavioral consequences of this mutation *in vivo*. Hence, we generated a line of *NLGN2* R215H knock-in (KI) mice using transgenic technology. Here, we report on the behavioral characterization of this line of mice.

## Materials and Methods

### Generation of *Nlgn2* R215H KI Mice and PCR Genotyping

The study was approved by the Institutional Animal Care and Use committee, Chang Gung University (Approval No.: CGU14-033). The transgenic mice were generated using the service of the Transgenic Mouse Model Core supported by National Core Facility Program for Biotechnology, National Science Council of Taiwan. In summary, a bacterial artificial chromosome (BAC) clone RP24-271B5 containing the mouse *Nlgn2* gene (strain C57BL/6) was purchased from the BACPAC resource center and was used for the construction of a targeting vector and mutagenesis following methods described previously (22). Exon 2 to exon 7 of the *Nlgn2* (12.8 kb in length) in the BAC clone was subcloned into vector pBluescript (containing HSV-TK gene for negative selection). The G-to-A mutation (R215H) was introduced into exon 3 by PCR-based mutagenesis. Further, a neomycin resistance cassette encompassed by two loxP sites was introduced into the mutant construct by recombination to generate the R215H gene targeting vector (R215H KI vector). The linearized R215H KI vector was transduced into embryonic stem cells (ESCs) of Jm8A mice (agouti C57BL/6N strain) (23) by electroporation. The ESC clones passing the positive and negative selections were introduced with a Cre recombinase expression vector *via* electroporation. The surviving ESC clones were subcultured and screened by Southern blot using a 5′ end external probe and internal probe that recognized correct restriction fragments, respectively. Finally, one of the KI ESC clones was selected for microinjection. The blastocysts were obtained from C57BL/6Nar1 mice, and the KI blastocysts were transferred into uteri of CD-1 surrogate mothers. The obtained chimera pups with high a percentage (>70%) of agouti fur were selected as founder for breeding. Since all the animal materials came from the C57BL/6 genetic background, the F1 heterozygotes carried pure C56BL/6N genetic background. Therefore, the intercross of heterozygotes was used to produce all three genotypes of animals for experiments (23). The pups were PCR genotyped using a pair of primers flanking the exon 4 (CU5:5′-ATAGCGGCCGCAGAGGATTGGTAGGGTCCAG-3′) and loxP site (FD5:5′-CACGTCGACTTAGTCCGCTCTCACCAGGA-3′). A PCR product of 1,000 bp indicates the R215H KI allele, while 900 bp indicates the control allele. All the animals were housed in groups of 4–6 per cage made of polycarbonate (29.6 cm long, 18.8 cm wide, 13.6 cm high) with free access to food and water. No special environmental enrichment was used. The animal room was controlled under a 12/12 h light–dark cycle (light from 0700 to 1900 hours). Room temperature was set at 22 ± 2°C, and humidity was set at 50 ± 10%. All the animals subjected to behavioral tests were older than 8 weeks, and both male and female of animals were tested. The behavioral tests were performed from 10:00 a.m. to 17:00 p.m. All experiments were performed in accordance with Taiwan animal protection laws.

### Physical, Biochemical, and Histological Examinations

Two representative animals (one male and one female) of each of three genotypes were sent to the National Laboratory Animal Center, Taiwan, for routine physical examination, blood biochemistry analysis, and histology examination. Body length, tail, and weight of three genotypes of animals were measured at the fifth, sixth, seventh, and eighth week after birth in the laboratory.

### Reverse Transcription and Quantitative PCR

Total RNA extracted from the brain tissue was subjected to reverse transcription using RevertAid H Minus First Strand cDNA Synthesis Kit (Thermo Fischer Scientific, MA, USA) according to the manufacturer’s protocol. The expression of the mutant cDNA was further verified using PCR-based sequencing with the primer pairs (*Nlgn2*-cDNA-F-Ex1: 5′-CTG CCT GTA CCT CAA CCT CT-3′ and *Nlgn2*-cDNA-R-Ex4: 5′-GCG CAG GGC CTG GAT CTG GT-3′).

### Western Blot Analysis

Western blot analysis was conducted using the protocol established in the laboratory with some modifications (24). In brief, brain tissue was homogenized in 500-μl cell lysis solution containing 5% sodium dodecyl sulfate (SDS), 50 mM Tris–HCl (pH 7.4), and 1 mM ethylenediaminetetraacetic acid per 50–100 mg tissue. The homogenates were centrifuged at 12,000 × *g* for 10 min at 4°C, and the supernatant was collected. The concentration of the total brain protein was measured using the Pierce BCA Protein Assay Kit (Thermo Fischer Scientific, MA, USA). A total of 30 µg of brain protein was separated on 7.5% SDS-PAGE gels at constant voltage (100 V) for 1 h, then transferred onto an Immobilon-P Membrane (polyvinylidene fluoride) for 2 h with constant voltage (100 V) at 4°C using Tank Transfer Systems (Mini Trans-Blot Cell, Bio-Rad Laboratories Inc., Hercules, CA, USA). After blocking with 10% skim milk for 1 h, the membrane was incubated with polyclonal rabbit antibody against *NLGN2* (1:1,000) (Synaptic Systems, Goettingen, Germany) at 4°C overnight with shaking. Goat anti-rabbit IgG conjugated with alkaline phosphatase (1:7,500) (Promega, Madison, WI, USA, Wisconsin) was used as the second antibody. Immunoreaction was detected by ProtoBlot II AP system (Promega, Madison, WI, USA). Image J was used to measure the intensity of the signal.

### Open Field Test

Locomotor activity of mice was assessed using the VersaMax Open Field Activity Monitoring system (AccuScan Instruments, Columbus, OH, USA). The activity chamber was made of Plexiglas with the size was 40 cm × 30 cm × 30.5 cm. Animals were placed in the cage for 1 h, and locomotor activity was recorded automatically. Total distance traveled and spent in the center and peripheral areas were analyzed by the software provided by the instrument. The order of testing of animals was random.

### Elevated Plus-Maze (EPM) Test

The test was conducted following the procedures described by Lin and colleagues in our institute (25). The EPM apparatus was made of black acrylic (polymethylmethacrylate). It consisted of two open arms and two closed arms. Each arm was 50 cm long and 10 cm wide. The maze was 50 cm above ground. The closed arms had additional clear acrylic walls of 40 cm high on both sides of the path. The apparatus was placed in a room illuminated with a white light of approximately 70 lx. The animal was placed on the center platform of the maze and was allowed to explore for 6 min. The order of testing of animals was random. The entry and the time of staying in open arms and closed arms of each animal were recorded and analyzed using the video-tracking system Ethovision (Noldus, Wageningen, The Netherlands).

### Prepulse Inhibition (PPI) Assay

Prepulse inhibition was conducted in an acoustic startle chamber (SR Lab, San Diego Instruments, CA, USA). The order of testing of animals was random. A continuous 65 dB SPL (decibel sound pressure level) background white noise within the chamber was maintained throughout the PPI tests. After being placed into the chamber for 5 min, each animal was presented with a series of six startle pulse-only (40-ms burst of 120 dB SPL) trials. This series of stimuli was followed by 60 randomized trials of five types: one trial was a pulse-only startle as described previously, another was no-pulse (no additional stimuli other than the white noise), and the remaining three involved various intensities of prepulse (20-ms burst of 69, 73, and 80 dB SPL), 100-ms preceding a 120-dB SPL startle pulse. These five types of trials were individually repeated 12 times in a pseudorandom way to make 12 blocks of PPI tests. Finally, another series of six startle pulse-only trials was conducted at the end of the experiment. The startle response was measured every 1 ms for a 100-ms period from the onset of the startle pulse stimulus, and the amplitude of the maximum response in each measurement was recorded for subsequent analyses. The percentage of PPI was calculated according to the following formula: % PPI = 100 − (P + S)/S × 100, where P + S is the recorded response amplitude for prepulse plus startle pulse trials and S is the recorded response amplitude for startle pulse-only trials.

### Morris Water Maze Test

The Morris water maze test was performed in a room evenly illuminated with a white light of approximately 70 lx. The water maze was a circular pool made of polymethylmethacrylate. It was 110 cm in diameter and 35.5 cm high. The water was prepared with skim milk and was kept at 21°C using ice. A white escape platform (8 cm in diameter, 10 cm high) was placed 1 cm below the water. The escape platform was placed at a constant quadrant (designated as zone 1) of the pool, not randomly allocated. The inner walls of four quadrants of the pool were decorated with four different geometric figures as cues for orientation. The swimming of the mice was monitored using the automated tracking system Ethovision (Noldus, Wageningen, The Netherlands). Over four consecutive days of training, each mouse was given four trials of swimming with a maximum duration of 2 min separated by a minimum of 15 min every day. If the mouse did not find the hidden platform, it was guided to the platform and allowed to remain on it for 30 s. On the fifth day, the hidden escape platform was removed, and a 90-s trial was performed for each mouse to test spatial memory. The proportion of time spent in each quadrant of the pool and the number of times the mouse crossed the former position of the hidden platform were recorded. The order of testing was random for each trial.

### Accelerated Rotarod Motor Test

A rotarod treadmill (Ugo Basile SPL, PA, USA) was used in this test. Mice were placed on the rotating rod for 5 min, initially with four revolutions per minute. From the 6th to the 10th minute, the rod accelerated from 4 to 40 revolutions. The latency until mice dropped from the rotating rod was recorded and averaged from three trials.

### Novel Location Recognition Test (NLRT) and Novel Object Recognition Test (NORT)

Tests were conducted following the procedures described by Lin and colleagues (26). In brief, both the NLRT and NORT were conducted in a Plexiglas open box (35 cm × 35 cm × 30 cm) located in a sound-attenuated room illuminated with a 20-W light bulb. After habituation in the test box 20 min per day for 2 days, on the third day, the animal was placed in the test box to freely explore for 5 min, then two identical objects were simultaneously placed into two corners. The animal was allowed to explore the two objects freely for 5 min, and the time of exploring each object was recorded. This session was defined as the sample phase. The exploring behavior was defined as when the animal’s head faced the object within approximately 1 cm distance or touched or sniffed the object. At the end of the sample phase, the animal was returned to the home cage immediately. After 30 min, the animal was tested for novel location recognition in the same test box with one of the two objects moved to a new corner. During the 5 min of testing, the time of exploring two objects was recorded. After 24 h, the same animal was tested in the NORT. The animal was tested in the same box with one of the two identical objects replaced with a novel object with different color and shape. The animal was tested for 5 min, and the time of exploring the original and the novel object was recorded. A preference index defined as the time of exploring the novel location or novel object over the total time of exploring both objects was calculated. The order of testing was random.

### Statistical Analysis

All the data were processed in Excel 2010 (Microsoft Corp., Seattle, WA, USA). All the interval data were expressed as mean ± SD. Ratio data were treated as interval data. Data from NLRT, NORT, EPM test, open field test, and accelerated rotarod test were analyzed by two-way analysis of variance (ANOVA) to address effects of sex, genotype, and their interaction. Comparisons between heterozygotes and homozygotes against control animals were analyzed by *post hoc* analysis of Dunnett’s test. Growth data from fifth to eighth week after birth, data from learning session of Morris water test, and PPI test were analyzed by two-way ANOVA with repeated measurements and multivariate analysis followed by *post hoc* analysis with Dunnett’s test. The data from the memory testing session were analyzed by two-way ANOVA followed by *post hoc* analysis with Dunnett’s test. *p* < 0.05 was considered statistically significant. All statistical tests were implemented using SPSS version 18.0 (IBM Corp., Armonk, NY, USA), and all graphical illustrations were generated in Excel 2010 (Microsoft Corp., Seattle, WA, USA).

## Results

### *Nlgn2* R215H Homozygous Mice Showed No Expression of *NLGN2* Protein

**Figure 1:**
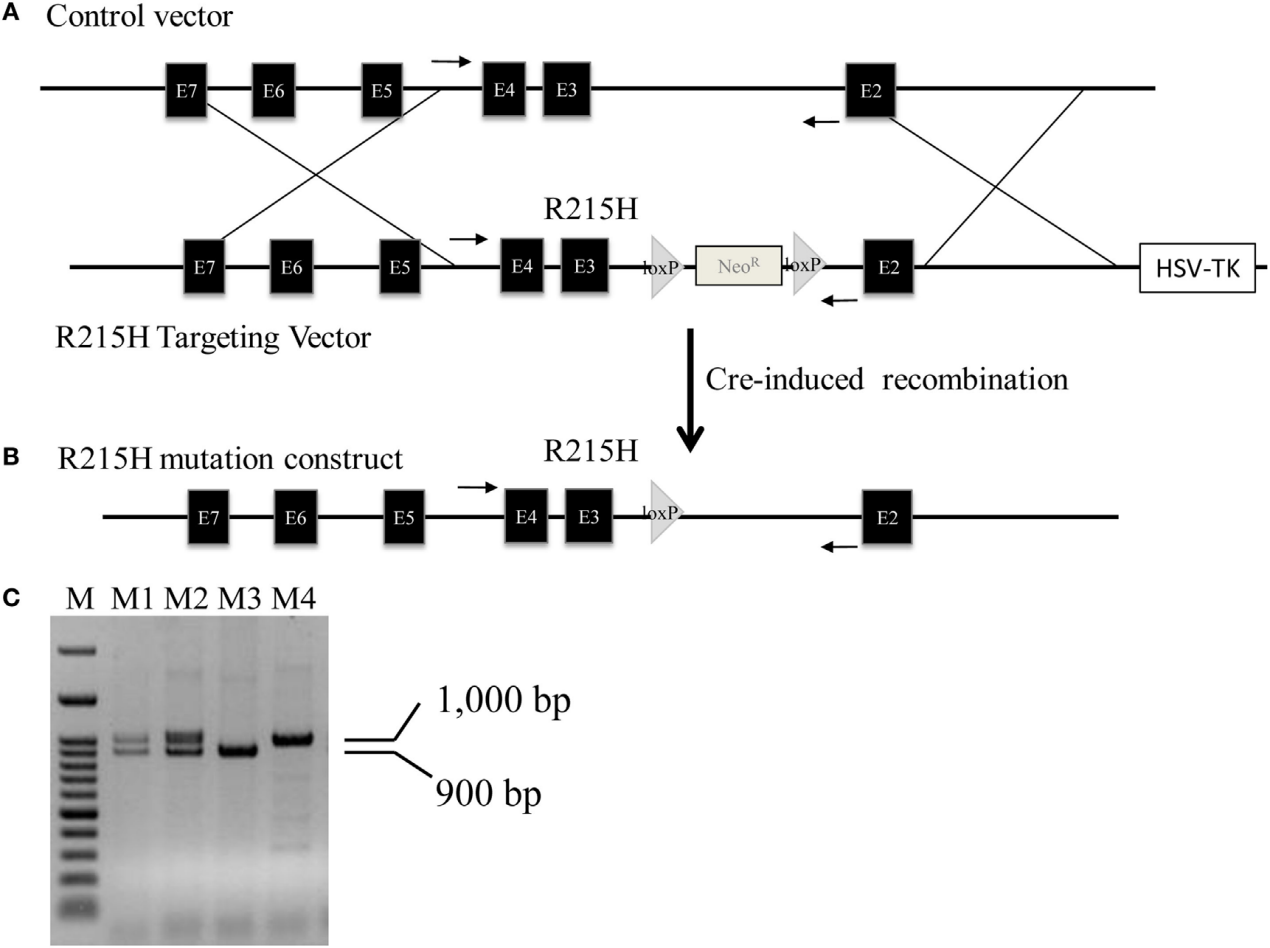
Generation of *Nlgn2* R215H knock-in (KI) mice. **(A)** Schematic constructs of *Nlgn2* control vector and R215H targeting vector. **(B)** Schematic construct of R251H KI mice. **(C)** PCR genotyping of mouse tail. M: 100 bp marker; M1 and M2: R215H heterozygotes; M3: control; M4: R215H homozygote. Arrows indicate the locations of PCR primers used for genotyping.

**Figure 2:**
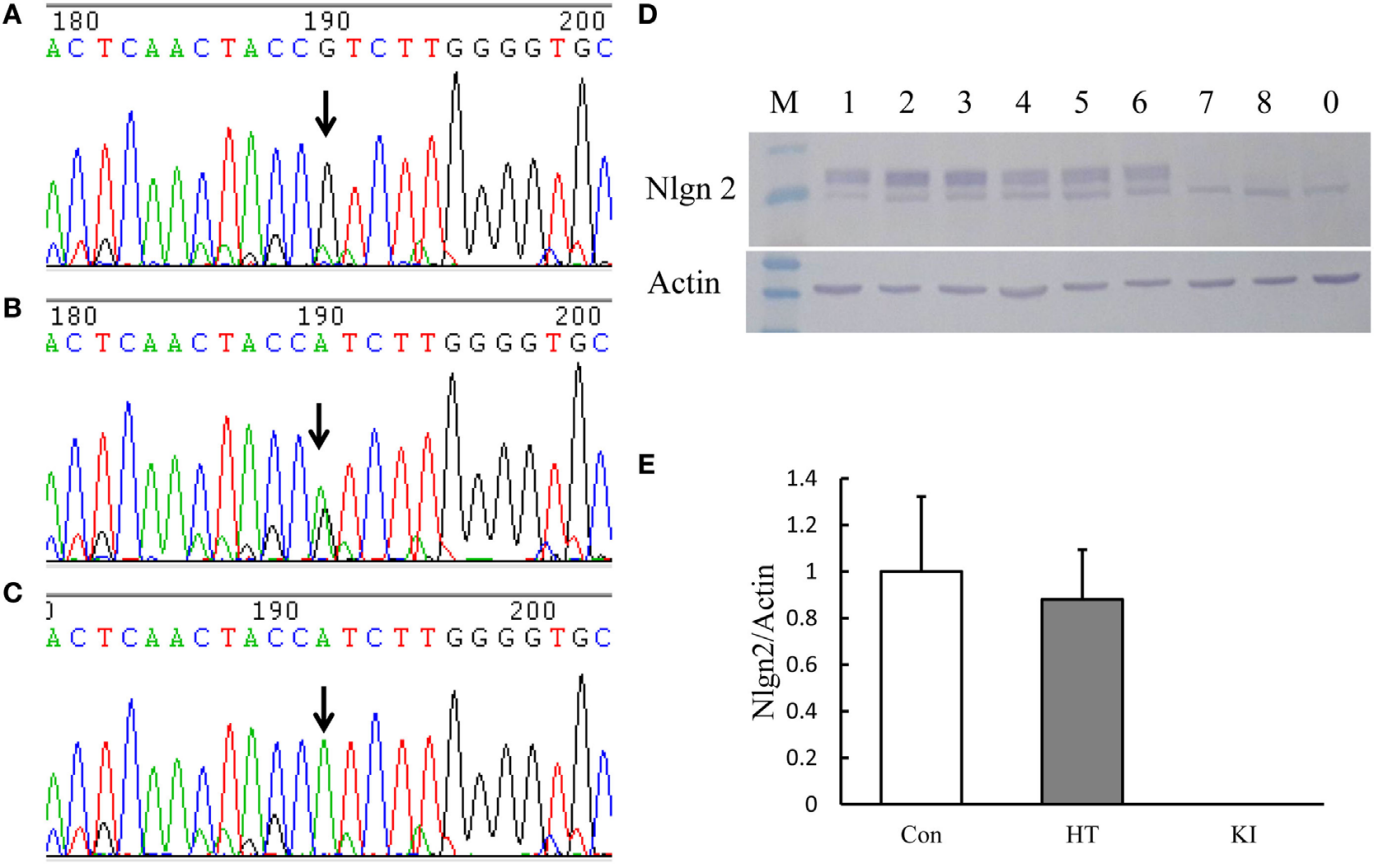
Analysis of the expression of *Nlgn2* R215H mutation in brain tissues of transgenic animals. **(A–C)** show the sequences of neuroligin 2 (*NLGN2*) cDNA expressed in the brain tissue of three genotypes of animals. Arrow indicates the site where the mutation sequence occurred. **(D)** Representative picture of Western blot analysis of *NLGN2* expression in the brain tissue of three genotypes of animals. **(E)**. Quantification of the Western blot analysis of *NLGN2* expressed in the brain of three genotypes of animals. Con indicates control animals (male, *n* = 5), HT indicates R215H knock-in (KI) heterozygous mice (male, *n* = 5), and KI indicates KI homozygous mice (male, *n* = 5).

We successfully generated a line of mice carrying the R215H mutation in the *Nlgn2* gene. Figure 1A shows the schematic constructs of control vector and the R215H targeting vector used to generate R215H KI mice. Figure 1B shows the schematic construct of R251H KI mice. Figure 1C shows genotypes of transgenic mice assessed by a PCR method. Expression of the R215H mRNA in transgenic mice was confirmed by sequencing the PCR products that covered the R215H mutation site using brain cDNAs as templates (Figures 2A–C). In further Western blot analysis, however, we found no expression of *NLGN2* protein in the brain of R215H homozygous mice (Figure 2D). In the brain of heterozygous mice (*n* = 5, males), expression of the *NLGN2* protein was reduced to approximately 88% of that in control animals (*n* = 5, males). The reduction did not reach statistical significance (*p* = 0.07) (Figure 2E).

### Delayed Postnatal Growth in R215H Homozygous Mice

**Figure 3:**
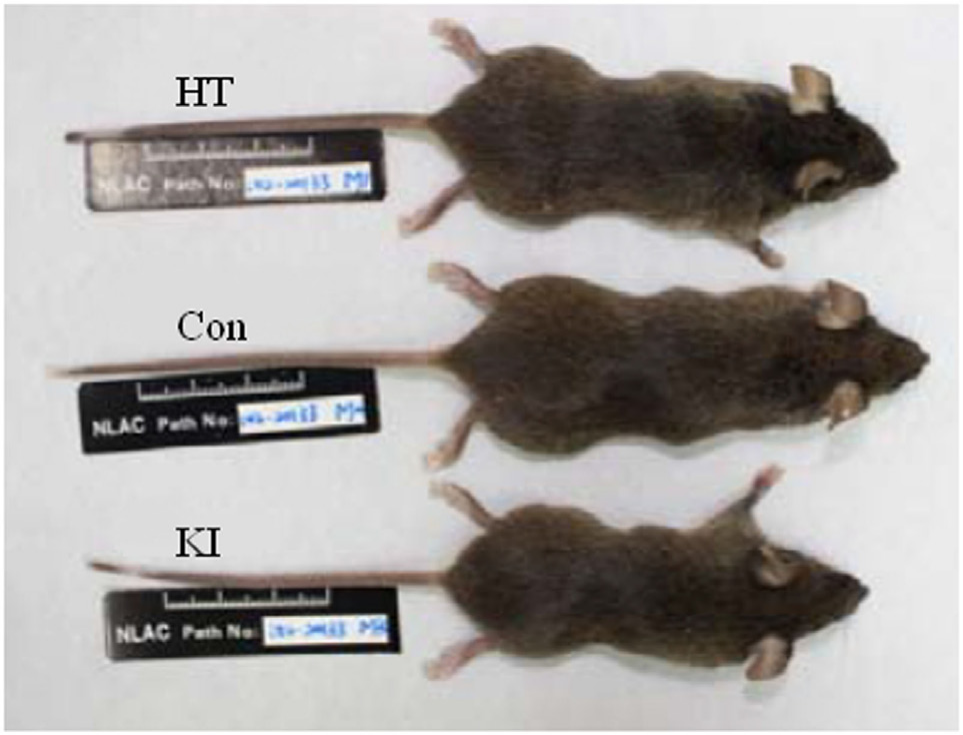
Pictures of three male genotypes of mouse. Con indicates control animal, HT indicates R215H knock-in (KI) heterozygote, and KI indicates KI homozygote.

**Figure 4:**
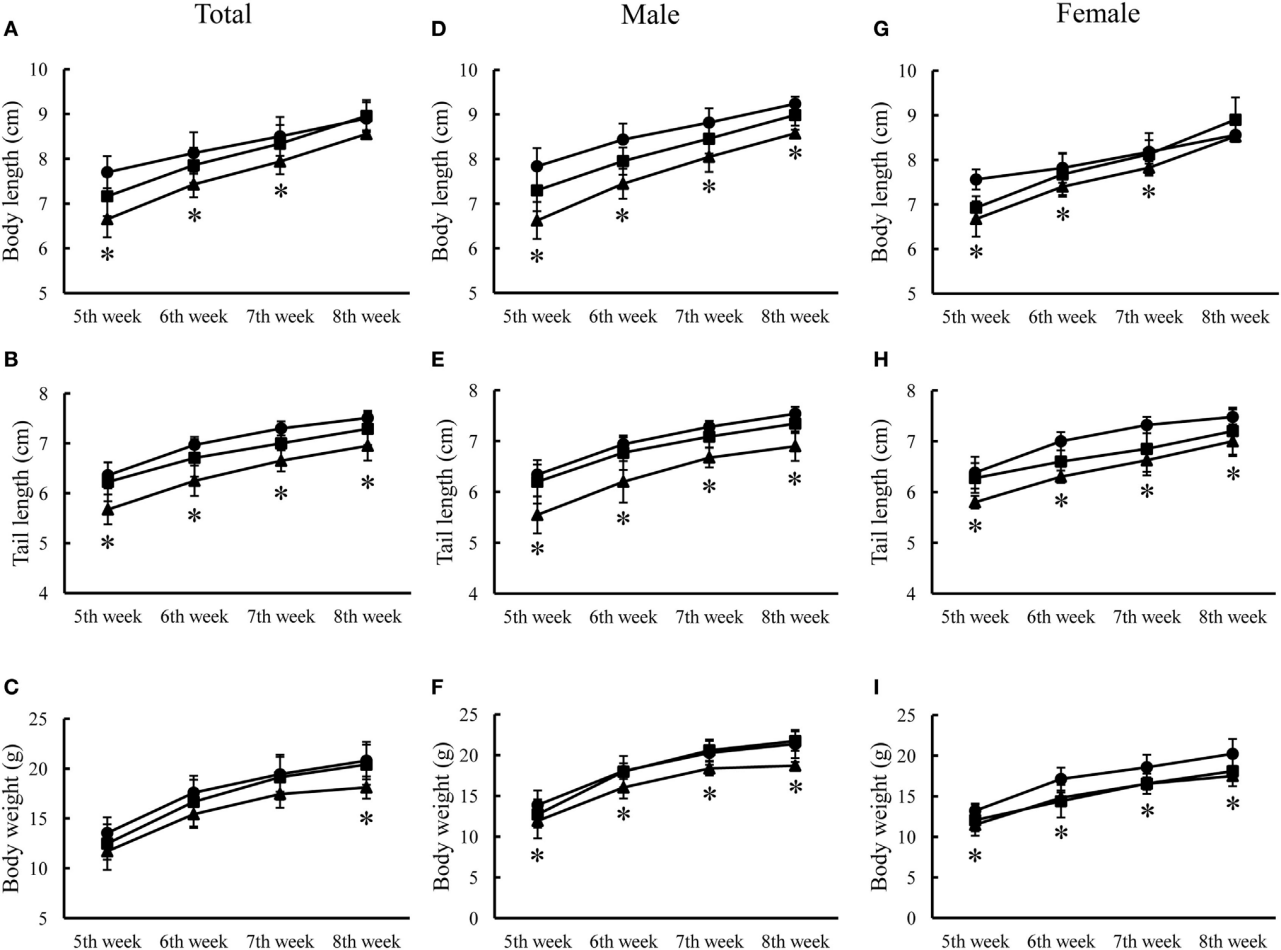
Postnatal growth curves of three genotypes of animals from the fifth week to eighth week after birth. **(A)** Growth curve of body length of three genotypes of animals. **(B)** Growth curve of tail length of three genotypes of animals. **(C)** Growth curve of body weight of three genotypes of animals. **(D–F)** are growth curves of body weight, body length, and tail length of male animals, respectively. **(G–I)** are growth curves of body weight, body length, and tail length of female animals, respectively. Black circle indicates control mice (*n* = 10, five males, five females); black square indicates heterozygous mice (*n* = 11, seven males, four females); black triangle indicates knock-in homozygous mice (*n* = 8, four males, four females) (*p < 0.05).

Two representative animals (one male and one female) of each of three genotypes of animals received routine examination of physical development, blood biochemistry, and histology examination from the National Laboratory Animal Center, Taiwan. No significant abnormalities were observed except that the R215H homozygous mouse was apparently smaller than the heterozygous and the control animals (Figure 3). Hence, we measured the postnatal growth curve of animals of three genotypes at the fifth, sixth, seventh, and eighth week after birth. In the analysis of body length, two-way ANOVA with repeated measurements revealed a significant correlation of body length between different weeks (*p* < 0.001). Significant main effects of sex (*F*_1, 23_ = 6.377, *p* = 0.019) and genotype (*F*_2, 23_ = 10.258, *p* = 0.001) on body length were observed, but there was no significant interaction between sex and genotype (*F*_2, 23_ = 1.401, *p* = 0.267). *Post hoc* analysis showed that homozygous mice (*n* = 8, four males, four females) were significantly shorter than control animals (*n* = 10, five males, five females) in body length at the fifth (*p* < 0.001), sixth (*p* = 0.003), and seventh (*p* = 0.015) week after birth. At the eighth week after birth, homozygous mice still had shorter body length compared to control mice, but the differences did not reach statistical significance (*p* = 0.06) (Figure 4A). When the animals were subgrouped by sex, both male and female homozygous mice showed significantly shorter body length than their respective controls, except at the eighth week, the differences in body length between female homozygous mice and female control did not reach statistical significance (*p* = 0.11) (Figures 4D,G).

In the analysis of tail length, two-way ANOVA with repeated measurements showed a significant correlation of tail length between different weeks (*p* < 0.001). A significant main effect of genotype (*F*_2, 23_ = 12.044, *p* < 0.001), but not sex (*F*_1, 23_ = 0, *p* = 0.997) on tail length was noted. There was no significant interaction between sex and genotype (*F*_2, 23_ = 0.356, *p* = 0.704). *Post hoc* analysis revealed that homozygous mice also had shorter tail length compared to control mice at the fifth (*p* = 0.001), sixth (*p* < 0.001), seventh (*p* < 0.001), and eighth (*p* = 0.001) week after birth, respectively (Figure 4B). When animals were subgrouped by sex, both male and female homozygous mice showed significantly shorter tail length than their respective controls from fifth week to eighth week after birth (Figures 4E,H).

In the analysis of body weight, two-way ANOVA with repeated measurements revealed a significant correlation of body weight between different weeks after birth (*p* < 0.001). Significant main effects of sex (*F*_1, 23_ = 11.554, *p* = 0.002) and genotype (*F*_2, 23_ = 5.631, *p* = 0.010) on body weight were observed, but there was no significant interaction between sex and genotype (*F*_2, 23_ = 1.493, *p* = 0.246). *Post hoc* analysis showed that there was a significantly lighter body weight in homozygous mice than control mice at the eighth week, but not at the fifth, sixth, and seventh week after birth (*p* = 0.01) (Figure 4C). When animals were subgrouped by sex, both male and female homozygous KI mice showed significantly lighter body weight than their respective controls at the eighth week, but not at the other weeks (Figures 4F,I). No significant differences in the postnatal growth curves were observed between R215H heterozygous mice (*n* = 11, seven males, four females) and control animals (*n* = 10, five males, five females).

### R215H Homozygous Mice Showed Enhanced Response in PPI Test

**Figure 5:**
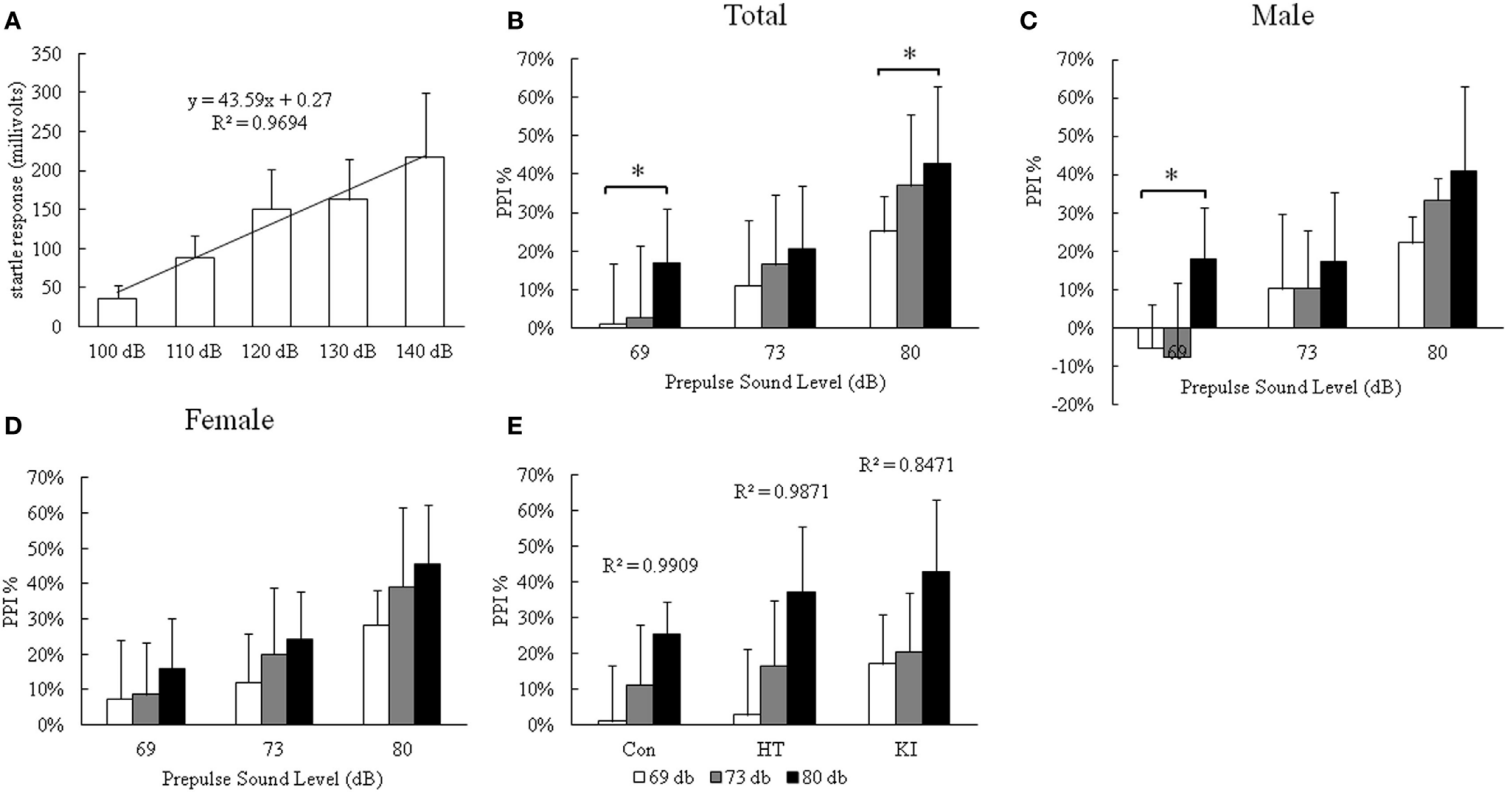
Results of prepulse inhibition (PPI) assay. **(A)** The dose–response curve of startle stimulus and startle response in four control mice (three male and one female). **(B)** PPI assay of three genotypes of animals. **(C)** PPI assay of three genotypes of male animals. **(D)** PPI assay of three genotypes of female animals. **(E)** Correlation of startle response with three different prepulse stimuli (*p < 0.05).

In the PPI test, we first demonstrated a dose–response relationship between startle stimulus and startle response from 110 to 140 dB in four control mice (three males and one female) (Figure 5A). There was a significant simple linear correlation between stimulus and startle response (*R*^2^ = 0.97). We chose 120 dB as the startle-inducing stimulus throughout the experiment. Two-way ANOVA with repeated measurement showed that there was a significant correlation of startle PPI between three prepulse intensities (*p* < 0.001). We observed a significant main effect of genotype (*F*_2, 33_ = 3.362, *p* = 0.047), but not sex (*F*_1, 33_ = 2.237, *p* = 0.144) on PPI. Also there was no significant interaction between sex and genotype (*F*_2, 33_ = 0.219, *p* = 0.805). Multivariate analysis revealed that R215H homozygous mice (*n* = 13, seven males, six females) showed significantly enhanced inhibition of startle response compared with control animals (*n* = 12, six males, six females) at the prepulse level of 69 dB (*p* = 0.035) and 80 db (*p* = 0.037), but not at 73 dB (*p* = 0.342) (Figure 5B). Although heterozygotes (*n* = 14, five males, nine females) also showed increased PPI compared with control animals, the differences did not reach statistical significance (69 dB, *p* = 0.944; 73 dB, *p* = 0.655; 80 dB, *p* = 0.178). When the animals were subgrouped by sex, no significant differences in PPI at three prepulse levels between homozygous and control animals were detected in both sexes, except that there was a significant enhanced PPI at the prepulse intensity of 69 dB in male homozygous mice compared to male control animals (*p* = 0.038) (Figures 5C,D). In addition, we also observed that PPI increased with increasing prepulse stimulus intensity in all three genotypes of mice (Figure 5E).

### R215H Homozygous Mice Showed Impaired Learning and Memory in Morris Water Maze Test

**Figure 6:**
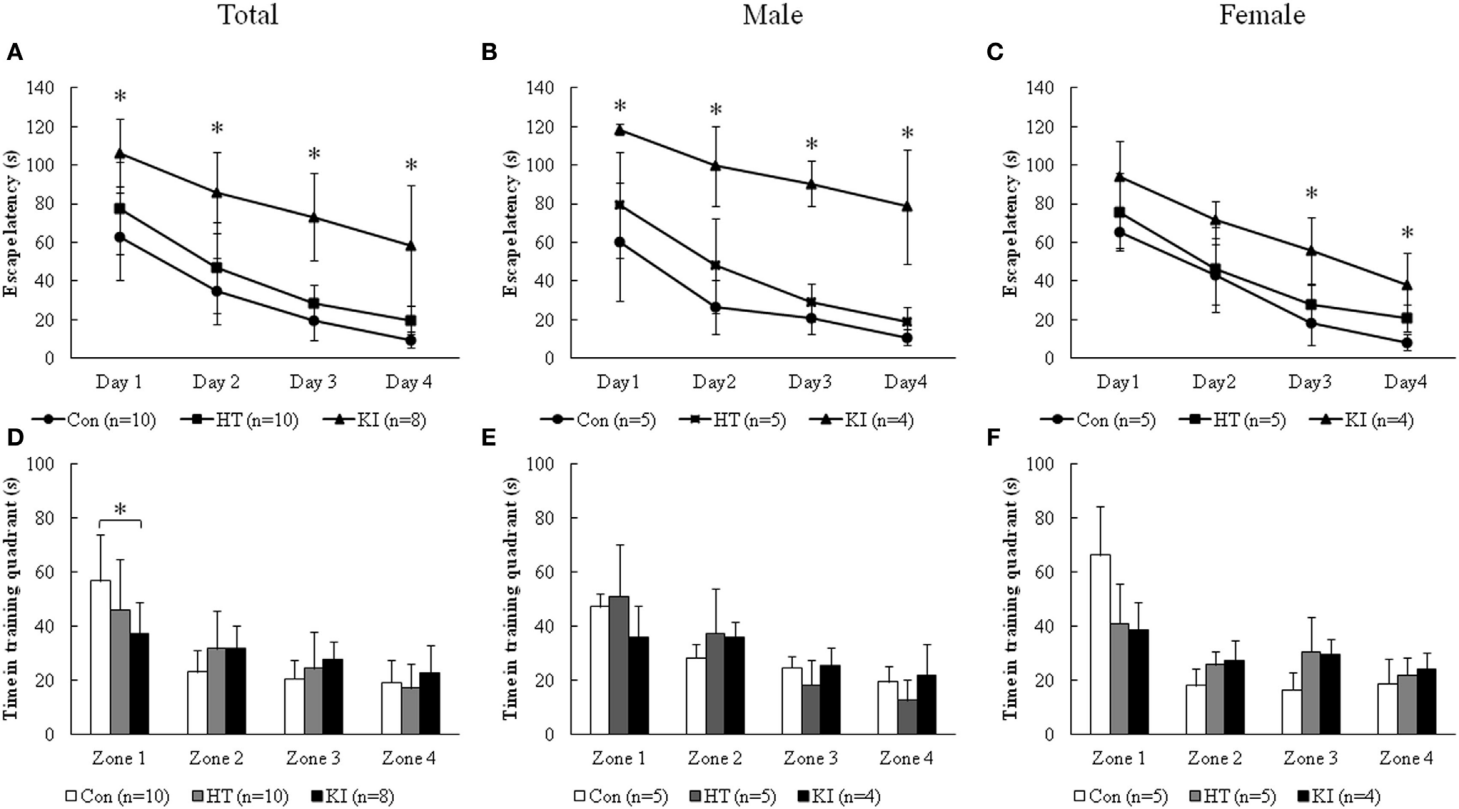
Morris water maze test. **(A)** Escape latency of three genotypes of animals during the 4 days of training. **(B)** Escape latency of three genotypes of male animals during the 4 days of training. **(C)** Escape latency of three genotypes of female animals during the 4 days of training. **(D)** Time of three genotypes of animals spent in four quadrants on the memory testing day. **(E)** Time of three genotypes of male animals spent in the four quadrants on the memory testing day. **(F)** Time of three genotypes of female animals spent in four quadrants on the memory testing day. Zone 1: the quadrant where the hidden platform was placed (*p < 0.05).

For the data from training session, two-way ANOVA with repeated measurements showed significant correlation of escape latency between 4 days of training (*p* < 0.001). Significant main effects of sex (*F*_1, 22_ = 7.162, *p* = 0.014) and genotype (*F*_2, 22_ = 65.134, *p* < 0.001) on the latency of finding the hidden platform were noted. In addition, there was a significant interaction between sex and genotype (*F*_2, 22_ = 9.159, *p* = 0.001). *Post hoc* analysis revealed significantly longer escape latency in homozygous mice than control animals from day 1 through day 4 (day 1, *p* = 0.001; day 2, *p* < 0.001, day 3, *p* < 0.001, day 4, *p* < 0.001) (Figure 6A). There were no differences in the escape latency between heterozygous animals and controls (day 1, *p* = 0.29; day 2, *p* = 0.38, day 3, *p* = 0.39, day 4, *p* = 0.38). When animals were subgrouped by sex, male homozygous animals showed significantly longer escape latency than control animals from day 1 to day 4, while female homozygous KI mice showed significantly longer escape latency than female control mice at day 3 and day 4, but not at day 1 and day 2 (Figures 6B,C).

In the analysis of the data of day 5 of memory testing, we found that control animals spent significantly more time in zone 1 where the platform was hidden than the other quadrants (zone 1 vs. zone 2, *p* < 0.001; zone 1 vs. zone 3, *p* < 0.001; zone 1 vs. zone 3, *p* < 0.001). Heterozygous and knock-in homozygous mice, however, did not show longer stay in zone 1 compared to the other quadrants. Two-way ANOVA revealed a borderline main effect of genotype (*F*_2, 22_ = 3.307, *p* = 0.055), but not sex (*F*_1, 22_ = 0.399, *p* = 0.534) on the time in zone 1. Also no significant interaction between sex and genotype (*F*_2, 22_ = 2.089, *p* = 0.148) was observed. *Post hoc* analysis showed that homozygous mice spent significantly less time in zone 1 compared to control animals (*p* = 0.034) (Figure 6D). When animals were subgrouped by sex, the differences of time in zone 1 between homozygous mice and control animals were not observed in both sexes of animals (Figures 6E,F). Also, we observed no main effects of sex, genotype, and their interaction on the time stayed in the other zones. No significant differences in retention time were observed between heterozygous and control mice (Figures 6D–F). During the experiments, we did not observe apparent physical or neurological deficits in the homozygous animals that may affect their swimming ability.

### Accelerated Rotarod Test

In the accelerated rotarod test, two-way ANOVA revealed no main effects of sex (*F*_1, 22_ = 0, *p* = 0.990) and genotype (*F*_2, 22_ = 1.005, *p* = 0.381), and no significant interaction between sex and genotype (*F*_2, 22_ = 0.125, *p* = 0.883). No significant differences in the retention time (seconds) were observed between R215H homozygous mice (153 ± 25, *n* = 8, four males, four females), heterozygous mice (174 ± 59, *n* = 10, five males and five females), and control animals (142 ± 49, *n* = 10, five males, five females).

### R215H Homozygous Mice Showed Impairments in NLRT but Not in Object Recognition Test (NORT)

**Figure 7:**
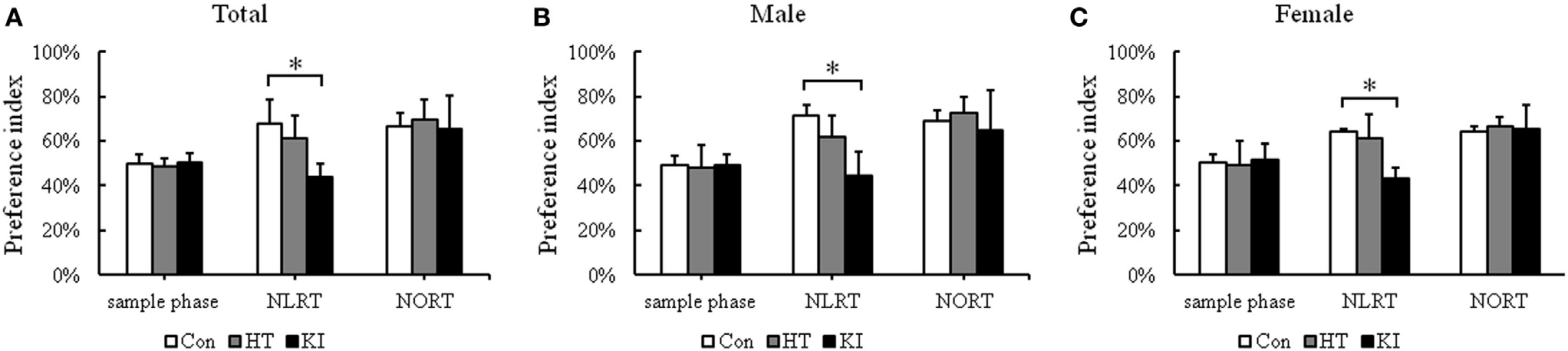
Results of novel location recognition test (NLRT) and novel object recognition test (NORT) **(A)** Comparison of preference index of three genotypes of animals in NLRT and NORT. **(B)** Comparison of preference index of three genotypes of male animals in NLRT and NORT. **(C)** Comparison of preference index of three genotypes of female animals in NLRT and NORT. Con, control animals; HT, heterozygotes; KI, knock-in homozygotes. *p < 0.05.

In the NLRT, two-way ANOVA revealed a significant main effect of genotype (*F*_2, 45_ = 27.447, *p* < 0.001), but not sex (*F*_1, 45_ = 1.259, *p* = 0.268). No significant interaction between sex and genotype (*F*_2, 45_ = 0.662, *p* = 0.521) was noted. *Post hoc* analysis showed that homozygous mice (*n* = 17, 10 males, 7 females) had significantly lower preference index than control animals (*n* = 17, 10 males, 7 females) (*p* < 0.001) (Figure 7A). When the animals were subgrouped by sex, both male (*p* < 0.001) and female (*p* = 0.002) homozygous mice showed significantly lower preference index in NLRT than their respective control animals (Figures 7B,C).

In the NORT, no significant main effects of sex (*F*_1, 45_ = 1.138, *p* = 0.292) and genotype (*F*_2, 45_ = 0.643, *p* = 0.531) were noted. Also there was no significant interaction between sex and genotype (*F*_2, 45_ = 0.366, *p* = 0.696) (Figure 7A). When animals were subgrouped by sex, there were no significant differences in NORT in both sexes (males, *p* = 0.7, females, *p* = 0.94) (Figures 7B,C).

Heterozygous mice (*n* = 17, 10 males, 7 females) showed no significant differences in the preference index in NLRT (*p* = 0.074) and NORT (*p* = 0.65) when compared with control animals (Figure 7A). Also, both sexes of heterozygous mice did not show significant differences in NLRT (males, *p* = 0.052; females, *p* = 0.8) and NORT (males, *p* = 0.79; females, *p* = 0.78) compared with their respective control animals (Figures 7B,C).

### R215H Homozygous Mice Showed Anxiety-Like Behavior in the Open Field Test

**Figure 8:**
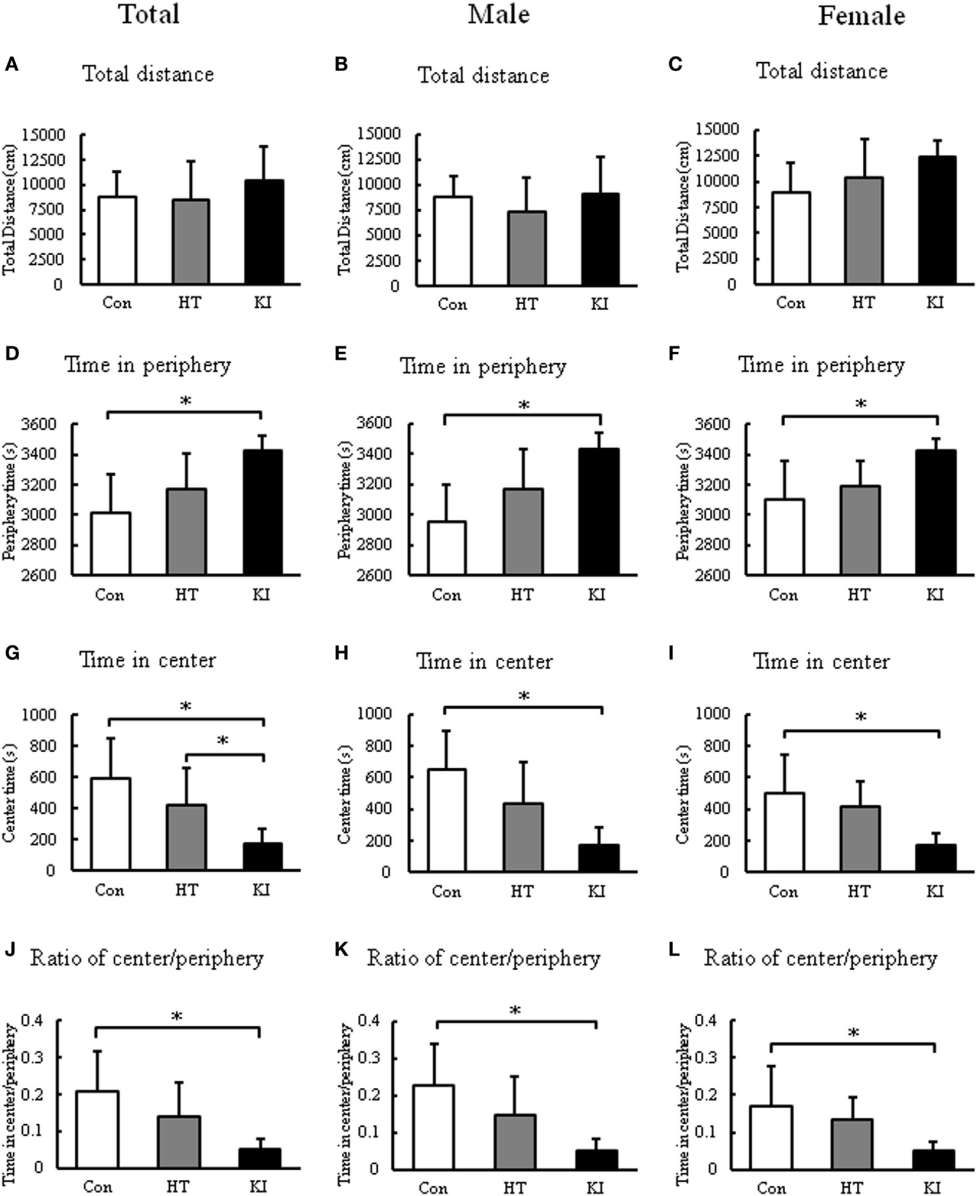
Locomotion analyses in the open field test. **(A)** Comparison of total distance moved of three genotypes of animals. **(B)** Comparison of total distance moved of three genotypes of male animals. **(C)** Comparison of total distance moved of three genotypes of female animals. **(D)** Comparison of time of three genotypes of animals spent in periphery of the arena. **(E)** Comparison of time of three genotypes of male animals spent in the periphery of the arena. **(F)** Comparison of time of three genotypes of female animals spent in the periphery of the arena. **(G)** Comparison of time of three genotypes of animals spent in the center of the arena. **(H)** Comparison of time of three genotypes of male animals spent in the center of the arena. **(I)** Comparison of time of three genotypes of female animals spent in the center of the arena. **(J)** Comparison of ratio of center/periphery of three genotypes of animals. **(K)** Comparison of ratio of center/periphery of three genotypes of male animals. **(L)** Comparison of ratio of center/periphery of three genotypes of female animals. Con, control animals; HT, heterozygotes; KI, knock-in homozygotes. *p < 0.05.

In the open field test, two-way ANOVA revealed a significant main effect of sex (*F*_1, 48_ = 5.884, *p* = 0.019), but not genotype (*F*_2, 48_ = 1.871, *p* = 0.165) on the total distance traveled. Also no significant interaction between sex and genotype (*F*_2, 48_ = 1.156, *p* = 0.323) was noted. *Post hoc* analysis revealed no differences in the total distance traveled between three genotypes. When the animals were subgrouped by sex, we still did not observe significant differences in the total distance traveled between three genotypes in both sexes (Figures 8A–C).

Nevertheless, we observed a significant main effect of genotype (*F*_2, 48_ = 15.101, *p* < 0.001), but not sex (*F*_1, 48_ = 0.981, *p* = 0.327) on the time spent in periphery. No significant interaction between sex and genotype was noted (*F*_2, 48_ = 0.599, *p* = 0.554). *Post hoc* analysis revealed that homozygous mice (*n* = 18, 11 males, 7 females) spent significantly more time in the periphery than control animals (*n* = 18, 11 males, 7 females) (*p* < 0.001) (Figure 8D). When animals were subgrouped by sex, both male (*p* < 0.001) and female (*p* = 0.01) homozygous mice spent significantly more time in periphery than their respective controls (Figures 8E,F).

In addition, two-way ANOVA revealed a significant main effect of genotype (*F*_2, 48_ = 15.101, *p* < 0.001), but not sex (*F*_1, 48_ = 0.984, *p* = 0.326) on the time spent in center. No significant interaction between sex and genotype was noted (*F*_2, 48_ = 0.599, *p* = 0.554). *Post hoc* analysis revealed that homozygous mice (*n* = 18, 11 males, 7 females) spent significantly less time in center than control animals (*n* = 18, 11 males, 7 females) (*p* < 0.001) (Figure 8G). When animals were subgrouped by sex, both male (*p* < 0.001) and female (*p* = 0.01) homozygous mice spent significantly less time in center than their respective controls (Figures 8H,I).

We further compared the ratio of time in center/periphery between three groups. Two-way ANOVA showed a significant main effect of genotype (*F*_2, 48_ = 12.341, *p* < 0.001), but not sex (*F*_1, 48_ = 1.032, *p* = 0.315). No significant interaction between sex and genotype was noted (*F*_2, 48_ = 0.530, *p* = 0.592). *Post hoc* analysis revealed that homozygous mice (*n* = 18, 11 males, 7 females) had significantly lower ratio of time in the center/periphery than control animals (*n* = 18, 11 males, 7 females) (*p* < 0.001) (Figure 8J). When the animals were subgrouped by sex, both male (*p* < 0.001) and female (*p* = 0.021) homozygous mice showed significantly lower ratio of time in center/periphery than their respective controls (Figures 8K,L). Heterozygous animals (*n* = 18, 11 males, 7 females) showed no significant differences in these parameters compared to control animals.

### R215H Homozygous Mice Showed Anxiety-Like Behavior in EPM Test

**Figure 9:**
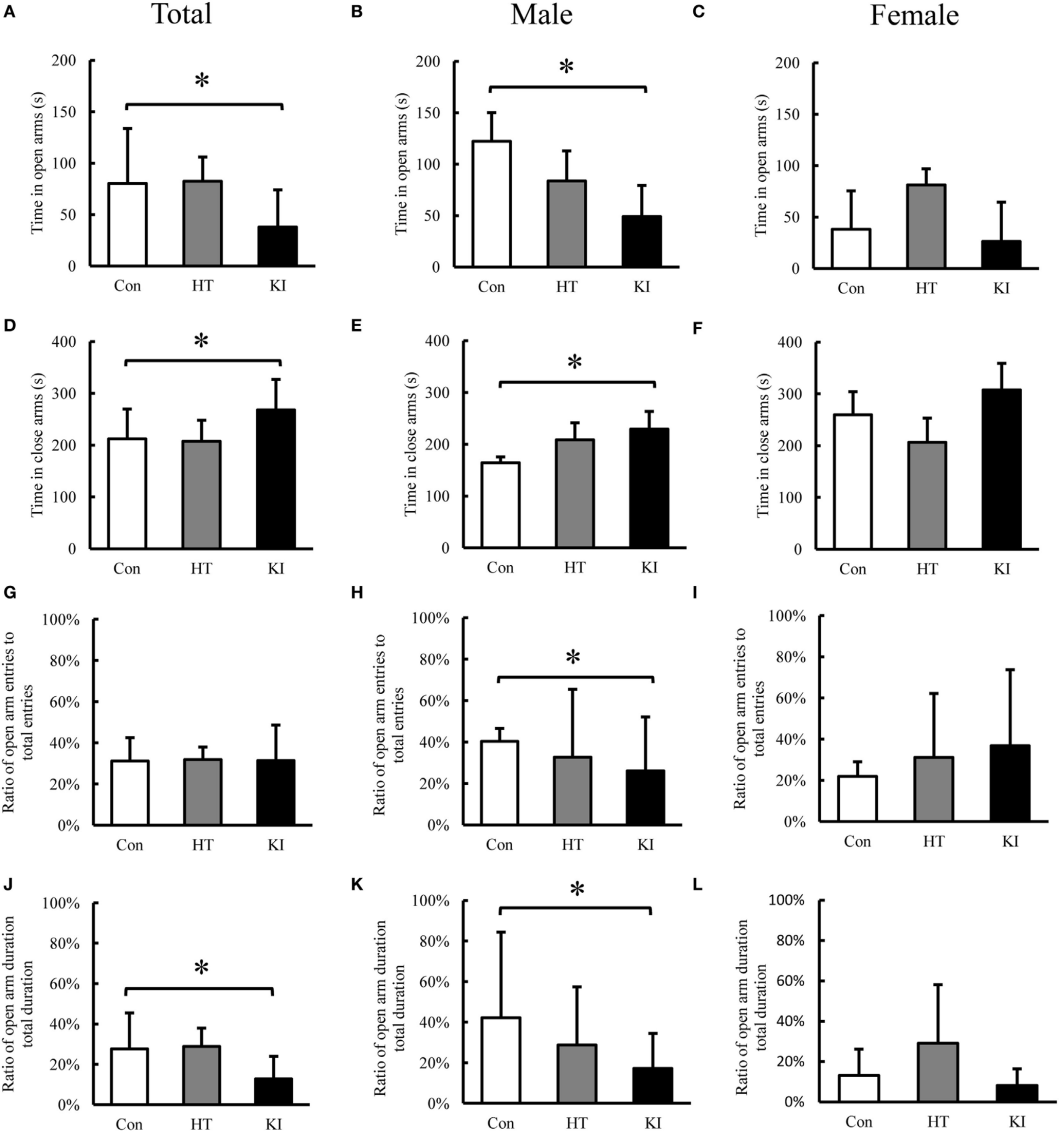
Behavioral analysis of the elevated plus-maze test. **(A)** Comparison of total time spent in open arms between three genotypes of animals. **(B)** Comparison of total time spent in open arms between three genotypes of male animals. **(C)** Comparison of total time spent in open arms between three genotypes of female animals. **(D)** Comparison of total time spent in closed arms between three genotypes of animals. **(E)** Comparison of total time spent in closed arms between three genotypes of male animals. **(F)** Comparison of total time spent in closed arms between three genotypes of female animals. **(G)** Comparison of ratio of open arm entry to total entry between three genotypes of animals. **(H)** Comparison of ratio of open arm entry to total entry between three genotypes of male animals. **(I)** Comparison of ratio of open arm entry to total entry between three genotypes of female animals. **(J)** Comparison of ratio of duration in open arms to total duration in open and closed arms between three genotypes of animals. **(K)** Comparison of ratio of duration in open arms to total duration in open and closed arms between three genotypes of male animals. **(L)** Comparison of ratio of duration in open arms to total duration in open and closed arms between three genotypes of female animals. Con, control animals; HT, heterozygotes; KI, knock-in homozygotes. *p < 0.05.

In the EPM test, two-way ANOVA revealed significant main effects of sex (*F*_1, 24_ = 8.423, *p* = 0.008) and genotype (*F*_2, 24_ = 3.830, *p* = 0.012) on time in open arms. Also a significant interaction between sex and genotype (*F*_2, 24_ = 15.101, *p* = 0.036) was noted. *Post hoc* analysis showed that homozygous mice (*n* = 10, five males, five females) spent less time in open arms compared to control animals (*n* = 10, five males, five females) (*p* = 0.02) (Figure 9A). When animals were subgrouped by sex, the differences of time in open arms were present in male homozygous mice (*n* = 5) compared to control male mice (*n* = 5) (*p* = 0.008) (Figure 9B), but not in female homozygous mice (*n* = 5) compared to female control mice (*n* = 5)(*p* = 0.075) (Figure 9C).

In the analysis of time in closed arms, two-way ANOVA also revealed significant main effects of sex (*F*_1, 24_ = 12.752, *p* = 0.002) and genotype (*F*_2, 24_ = 5.978, *p* = 0.008) on time in closed arms. Also a significant interaction between sex and genotype was noted (*F*_2, 24_ = 3.500, *p* = 0.046). *Post hoc* analysis showed that homozygous mice (*n* = 10, five males, five females) spent significantly more time in closed arms compared to control animals (*n* = 10, five males, five females) (*p* = 0.016) (Figure 9D). When animals were subgrouped by sex, male homozygous mice (*n* = 5) spent significantly more time in closed arms than male control animals (*n* = 5) (*p* = 0.013) (Figure 9E), but this phenomenon was not observed in female homozygous mice (*n* = 5) compared to female control mice (*n* = 5) (*p* = 0.299) (Figure 9F).

In the analysis of ratio of open arm entry/total entry (open arm entry plus closed arm entry), two-way ANOVA revealed no main effects of sex (*F*_1, 24_ = 0.500, *p* = 0.486) and genotype (*F*_2, 24_ = 0.011, *p* = 0.989), and a significant interaction between sex and genotype was observed (*F*_2, 24_ = 3.719, *p* = 0.039). *Post hoc* analysis revealed no significant differences between three genotypes (Figure 9G). When animals were subgrouped by sex, male homozygous animals showed significantly lower ratio of entry compared to male control animals (*p* = 0.009) (Figure 9H). No significant differences were observed between female homozygous KI mice compared with female control animals (*p* = 0.263) (Figure 9I).

In the analysis of ratio of duration in open arm/total duration, two-way ANOVA revealed significant main effects of sex (*F*_1, 24_ = 9.648, *p* = 0.005) and genotype (*F*_2, 24_ = 6.585, *p* = 0.005), and a significant interaction between sex and genotype (*F*_2, 24_ = 4.588, *p* = 0.021). *Post hoc* analysis showed that homozygous mice had significantly lower ratio compared to control animals (*p* = 0.012) (Figure 9J), When animals were subgrouped by sex, male homozygous animals showed significantly lower ratio compared to male control animals (*p* = 0.003) (Figure 9K); however, this phenomenon was not observed in female homozygous animals when compared to female control animals (*p* = 0.053) (Figure 9L).

## Discussion

In this study, we successfully created a line of *NLGN2* R215H mutant mice using a recombinant-based method (22). In Western blot analysis of mutant brain tissues, surprisingly, we detected no expression of the mutant protein in homozygous mutant mice. To understand the possible mechanism, we extracted total RNA from the brain tissue of all three genotypes and conducted reverse transcription and PCR-based sequencing. We found that the R215H mRNA mutation was expressed in both heterozygous and homozygous mutant animals. Furthermore, we overexpressed the *Nlgn2* cDNA containing the R215H mutation in HEK293 cells, and conducted Western blot analysis. This mutant protein was expressed in the HEK293 cells, which was consistent with our previous publication (21). In that study, we demonstrated that the R215H protein was trapped in the endoplasmatic reticulum and was not translocated to the cell membrane (21). Hence, there is a discrepancy in the expression of *NLGN2* R215H mutation in live animals and in the overexpression of this mutation in cell culture at the protein level. The reason for this discrepancy needs further study in the future. The absence of *NLGN2* expression in the R215H KI mice resembles the effect of *Nlgn2* knockout in mice.

The mutant mice were viable, fertile, and had an unremarkable appearance except that homozygous mutant mice were physically smaller than control animals. Further assessment revealed that the homozygous mutant mice had a significant delay in growth curve compared to control animals. The growth retardation of R215H *Nlgn2* KI homozygous mice found in this study is compatible with the findings of *Nlgn2* knockout mice generated by Wohr and colleagues (27). In their study, they reported delays in physical development and early growth milestones in the *Nlgn2* knockout mice, such as body length (but not body weight), tail length, eye-opening, incisor eruption, and grasp reflex. Taken together, our results suggest that *NLGN2* may play a role in the physical growth of mice.

Besides growth retardation, R215H homozygous mice were found to have increased anxiety-like behavior in the open field test and in the EPM test. The anxiety-like behaviors are also in line with the findings in the *Nlgn2* knockout mice reported by Blundell and colleagues (28). In their study, they found a marked increase in anxiety-like behavior, a decrease in pain sensitivity, and a slight decrease in motor coordination in the *Nlgn2*-deficient mice (28). Furthermore, Babaev and colleagues also reported that *Nlgn2* knockout mice showed a robust anxiety phenotype in all three anxiety tests, including the EPM, the open field test, and the light/dark exploration test. Additionally, *Nlgn2* knockout mice showed reduced locomotor activity and enhanced freezing specifically under anxiogenic conditions (29). Taken together, these findings indicate that dysfunction of *NLGN2* contributes to the genesis of anxiety-like behaviors.

Notably, we found that R215H homozygous mice showed impairments in the NLRT, but not in NORT. In addition, R251H homozygous mice showed deficits in learning sessions and the memory session in Morris water maze test. Homozygous mice had similar learning curve to that of control mice; however, they were significantly slower than those of control animals, which might be due to their impaired spatial memory as indicated in the memory testing of Day 5. Together these findings suggest that *NLGN2* protein is associated with spatial learning and memory function in mice. Our findings are partly compatible with the study reported by Liang and colleagues (30). In their study, they conducted conditional knockout of *Nlgn2* in the medial prefrontal cortex of adult mice and found that the conditional knockout mice showed less anxiety-like behavior, which is opposite to our finding in our animals. However, the mice had significantly decreased contextual and cued fear conditioning, reflecting that *Nlgn2* knockout mice had defective learning and memory function (30). Together, these data suggest that dysfunction of *NLGN2* also contributes to impaired cognitive function.

In the PPI test, we found that the R215H homozygous KI mice had augmented inhibition of acoustic startle response compared to the control animals, which is an unusual finding. In the literature, most studies reported deficient PPI rather than enhanced PPI associated with neuropsychiatric disorders (31, 32). In human, to our knowledge, only cocaine abusers were reported to have enhanced PPI rather than attenuated PPI. Preller and colleagues reported that both pure recreation users and dependent cocaine users showed increased PPI of the acoustic startle response (33). PPI of the acoustic startle response reflects sensorimotor gating and involves neural circuits in several parts of the brain, including the prefrontal cortex, nucleus accumbens, ventral pallidum, and pontine tegmentum (34, 35). In addition, studies showed that neural circuits of sensorimotor gating are regulated by catecholamine neurotransmission in these parts of the brain (36, 37). The underlying mechanism of enhanced PPI in R215H homozygous mice is unclear at this moment and needs more studies in the future. In this study, we showed a dose–response correlation between startle stimulus and startle response from 110 to 140 dB in four control mice. Due to logistic problem, we were not able to perform this experiment in transgenic animals at this moment. As the baseline startle response is important for comparison between three genotypes of animals, this is a limitation of this study.

In this study, we used two-way ANOVA to examine the main effects of sex and genotype and their interactions on behavioral tests. The data were summarized in Table 1. Genotype has a significant main effect in the majority of the behavioral tests, except for accelerated rotarod test, novel objet recognition test, and total distance traveled in open field test. Sex has a significant main effect on body length, body weight, learning session of Morris water maze test, total distance traveled in Open field test, and EPM test. In most tests, there was no significant interaction between sex and genotype, except for learning session of Morris water maze test and EPM test. However, these results are not conclusive, as the current study is limited by the small sample size of mice used in this study, especially when the animals were subgrouped by sex. Therefore, the interpretation of sex differences in the behavioral tests of the R215H homozygous mice needs to be cautious. In addition, the study lacks biochemical and electrophysiological data to correlate with behavioral observations, which needs to be studied further in the future.

**Table 1:** Results of main effects of genotype and sex and interaction between genotype and sex in this study.

	Genotype	Sex	Genotype × Sex
Body length	+	+	−
Tail length	+	−	−
Body weight	+	+	−
Prepulse inhibition test		−	−
Morris water maze test—learning		+	+
Morris water maze test—memory		−	−
Acclerated rotarod test	−	−	−
Novel location recognition test		−	−
Novel object recognition test	−	−	−
Open field test—total distance traveled	−		−
Open field test—time in periphery		−	−
Open field test—time in center		−	−
Ratio of time in center/periphery		−	−
Elevated plus-maze (EPM)—time in open arms		+	+
EPM—time in closed arms		+	+
EPM—ratio of open arm entry/total entry		+	+
EPM—ratio of duration in open arm/total duration		+	+

*+ indicates presence of significant effect, − indicates absence of significant effect*.

In summary, we generated a line of *Nlgn2* R215H KI mice in this study. The mutant mice did not express the mutant protein in the brain, resulting in effects similar to *Nlgn2*-knockout mice. The animals expressed increased anxiety-like behaviors, aberrant sensorimotor gating function, and impaired spatial learning and memory, which resemble several clinical symptoms of psychiatric disorders. The study suggests that dysfunction of *NLGN2* contributes to the pathogenesis of several psychiatric symptoms in patients with mental illnesses. Thus, the *Nlgn2* R215H homozygous mice can be used as an animal model for psychiatric research.

## Ethics Statement

The study was approved by the Institutional Animal Care and Use committee, Chang Gung University (Approval No.:CGU14-033).

## Author Contributions

C-HC designed, supervised, analyzed the data, and wrote the manuscript. P-WL and H-ML conducted the experiments. P-KC conducted the required experiments and reanalyzed the data during revision. All the authors reviewed and approved the manuscript.

## Conflict of Interest Statement

The authors declare that the research was conducted in the absence of any commercial or financial relationships that could be construed as a potential conflict of interest.
